# Identification of a novel HIV-1 circulating recombinant form (CRF209_cpx) and its descendant unique recombinant form (URF) CRF209_cpx/B among MSM in Guangdong, southern China

**DOI:** 10.1186/s12985-026-03237-8

**Published:** 2026-06-28

**Authors:** Yaqing Lin, Liting Zhang, Haohui Deng, Pinfu Lai, Linna Liu, Xuemei Ling, Peng Qian, Haisheng Yu, Linghua Li, Ruolei Xin, Yun Lan

**Affiliations:** 1 Institute of Infectious Diseases and Guangzhou Key Laboratory of Clinical Pathogen Research for Infectious Diseases, Guangzhou Eighth People’s Hospital, Guangzhou Medical University, Guangzhou, 510000 China; 2 Department of Infectious Disease, The Fifth People’s Hospital of Shunde District, Longjiang Hospital of Shunde District, Foshan City, Foshan City), Foshan, 528000 China; 3 Infectious Disease Center, Guangzhou Eighth People’s Hospital, Guangzhou Medical University, Guangzhou, 510000 China; 4 Guangdong Center for Diagnosis and Treatment of AIDS, Guangzhou, 510000 China; 5 Institute of AIDS/STD Prevention and Control, Beijing Center for Disease Prevention and Control, Beijing, 100013 China

**Keywords:** HIV, Unique Recombinant Form (URF), Circulating Recombinant Form (CRF), CRF209_cpx, Phylogenetic Inference

## Abstract

**Background:**

The epidemic of human immunodeficiency virus type 1 (HIV-1) continues to pose a significant global health challenge, with increasing genetic diversity. The co-circulation of multiple subtypes among the local population facilitates the emergence of unique or circulating recombinant forms (URFs or CRFs). In China, the predominant strains include CRF07_BC, CRF01_AE, CRF55_01B, and subtype B. This study characterizes a novel CRF209_cpx and its descendant recombinant CRF209_cpx/B among men who have sex with men (MSM) in Guangdong, southern China.

**Methods:**

Individuals infected with URFs with similar genetic characteristics were recruited during routine surveillance of pretreatment drug resistance. Near full-length genomes (NFLGs) were amplified with two overlapping fragments using a serial dilution nested PCR approach after reverse transcription. We used SimPlot and IQ-TREE softwares to conduct recombination analyses and phylogenetic inferences. Time-scaled maximum clade credibility (MCC) phylogenetic trees were reconstructed using BEAST software to estimate evolutionary origins. Genotypic drug resistance mutations were interpreted via the Stanford HIV Database, and coreceptor usage was predicted using geno2pheno coreceptor 2.5 and the HIVcoPRED tool.

**Results:**

Four NFLG sequences were obtained and identified as a novel CRF209_cpx, generated by recombination among CRF01_AE, CRF07_BC and subtype B. Phylogenetic analyses revealed that all the parental segments clustered with lineages prevalent among MSM in China. Bayesian evolutionary analysis estimated that the most recent common ancestor (tMRCA) of CRF209_cpx to have evolved between 2011 and 2013. The fifth strain was identified as a URF recombined from nascent CRF209_cpx and B. No transmitted drug resistance mutation was detected in these five sequences. The four CRF209_cpx sequences primarily utilized the CXCR4 coreceptor, while the URF exhibited R5/X4 dual tropism.

**Conclusions:**

The emergence of the complex CRF209_cpx and novel URF of CRF209_cpx/B highlights the active HIV-1 epidemic within the MSM population in Guangdong, underscoring the necessity for enhanced molecular surveillance and precise public health intervention in this key population.

**Supplementary Information:**

The online version contains supplementary material available at https://doi.org/10.1186/s12985-026-03237-8.

## Introduction

 Human immunodeficiency virus type 1 (HIV-1) expresses high genetic diversity because of its error-prone replication and frequent recombination, which plays crucial roles in the rapid viral evolution and enormous variants [1]. To date, a total of 205 circulating recombinant forms (CRFs) that originated from the 10 subtypes (A-D, F-H, and J-L) and CRFs of HIV-1 Group M have been identified and registered [2], and numerous unique recombinant forms (URFs) have also been identified worldwide [3–4]. URFs are usually generated under the circumstance of superinfection with different subtypes or CRFs of inter-subtype recombination, or the different variants of intra-subtype recombination, otherwise co-infection with two distinct subtypes or CRSs. The further spread of the URFs across the population without obvious epidemiology relationship, resulting in the emergence of novel CRFs [4].

Over the past two decades, the top four subtypes or CRFs in China have evolved into CRF07_BC, CRF01_AE, CRF08_BC and subtype B, with the proportions of CRF07_BC and CRF01_AE have been gradually increasing since 2006 [5–6]. The co-circulation of multiple subtypes facilitates the emergence of URFs nationwide and, consequently, novel CRFs. To date, more than 70 CRFs have been identified in China, including CRF162_cpx reported by our team from recombination among CRF01_AE, CRF07_BC and subtype B in Guangdong Province [7]. The HIV-1 epidemic in Guangdong Province is characterized by high genetic diversity and numerous men who have sex with men (MSM) with active sexual contact [7–8].

Here, we identified and characterized another novel CRF composed of CRF01_AE, CRF07_BC and subtype B among newly diagnosed HIV-1-infected MSM in Guangdong, named after CRF209_cpx. Furthermore, a secondary third-generation URF derived from the recombination between nascent CRF209_cpx and subtype B has simultaneously been identified, which potentially indicates that CRF209_cpx expressed a local epidemic.

## Materials and methods

### Study population

Newly diagnosed HIV-1 individuals were recruited for routine surveillance of pretreatment drug resistance from 2018 to 2022 in Guangzhou, Guangdong. Five individuals were selected with discordant subtyping by *pol* gene protease-reverse transcriptase (PR-RT) and integrase (IN) fragments and expressed genetic similarity with tight phylogenetic cluster. They were all infected via MSM and without obvious epidemiological linkages. The basic demographic and clinical characteristics of the cases are listed in Table 1.

**Table 1:** Sociodemographic and clinical characteristics of five HIV-1 infected individuals

Sample ID	Gender	Age	Date at diagnosis	Date at sampling	Sampling City	Transmission Route	Baseline CD4 cell count (cells/µl)
ZLQ07878	Male	23	2022/1/18	2022/1/19	Guangzhou	MSM	360
16–057	Male	20	2018/9/9	2018/10/24	Guangzhou	MSM	377
220582	Male	25	2022/7/18	2022/7/18	Guangzhou	MSM	234
ZLQ03992	Male	22	2020/5/28	2020/7/3	Guangzhou	MSM	382
14–089	Male	25	2018/9/4	2018/9/14	Guangzhou	MSM	197

MSM: Men who have sex with men

### Nucleic acid extraction, amplification, sequencing, and sequence assembly

Viral RNA was extracted from the plasma sample using an automatic magnetic bead-based Virus RNA Extraction Kit (Daan Gene, China) and a semiautomatic Smart 32 nucleic acid extraction instrument (Daan, China). The RNA was subsequently reverse transcribed into cDNA with SuperScript III First-Strand Synthesis System (Invitrogen, United States) by two overlapping half genome fragments, and nested polymerase chain reaction (PCR) was performed using LA Taq (TaKaRa, China) with a serial dilution method as described previously [7]. Positive products were directly purified and sequenced by Sangon Biotech (Shanghai) Co., Ltd, using the Sanger sequencing method. The NFLGs were assembled and cleaned by the software Sequencher V5.1 (Gene Codes, United States). The obtained NFLG sequences were examined using the Quality Control Tool in the HIV database (https://www.hiv.lanl.gov/content/sequence/QC/index.html) to assess potential contamination and confirm sequence quality.

### Phylogenetic and recombination analysis

The maximum likelihood (ML) phylogenetic tree of the NFLG sequences was constructed using IQ-TREE (https://www.hiv.lanl.gov/content/sequence/IQTREE/iqtree.html) with 1000 bootstrap replicates. The best-fit nucleotides substitution model was chosen using ModelFinder. The subtyping reference sequences were downloaded from the Los Alamos National Library (LANL) HIV database (https://www.hiv.lanl.gov/content/index), including 10 subtypes (A-D, F–H, and J-L), CRF01_AE, CRF07_BC, and the secondary CRFs composed of CRF01_AE and CRF07_BC reported in China.

Preliminary analyses of recombination patterns were conducted using the online tool Recombinant Identification Program (RIP) and jpHMM from the LANL HIV sequence database with a window size of 400 bp. Similarity and BootScan analyses were performed using SimPlot software V3.5 [9] to further investigate the statuses of the recombinant forms.

The genome mosaic maps of the recombination pattern were drawn with the Recombinant HIV-1 Drawing Tool according to the positions of the reference HXB2 nucleotides in the LANL HIV Sequence Database (https://www.hiv.lanl.gov/content/sequence/DRAW_CRF/recom_mapper.html).

ML phylogenetic trees for subregions were constructed using IQtree with the best-fit nucleotides substitution model and 1000 ultrafast bootstrap replicates, alongside reference sequences included CRF01_AE clusters C1-C11, CRF07_BC clusters O and N, and Chinese B’ sequences [10–11]. The best-fit nucleotide substitution models chosen and applied for the ML phylogenetic trees reconstruction using IQ-TREE in this study are summarized in Supplementary Table 1.

### Bayesian evolutionary analysis and the prevalence of CRF209_cpx

To estimate the time of the most recent common ancestor (tMRCA) of CRF209_cpx, Bayesian molecular clock analyses were performed on the concatenated recombinant segments of each parental strain, using the Markov Chain Monte Carlo (MCMC) inference implemented in BEAST software V1.10 [12]. The MCMC evolutionary analysis was run for 200 million steps, with sampling every 5000 steps, under parameters of GTR nucleotide substitution model, uncorrelated relaxed clock model, and Bayesian skyline prior. The reference sequences of CRF01_AE, CRF07_BC and subtype B were downloaded from the LANL HIV database and selected to reconstruct the topology of the maximum clade credibility (MCC) trees. The log files were interpreted using Tracer v1.7.1 (effective sample size > 200 for all parameters) [13]. The initial 10% of states from each run were discarded as burn-in using TreeAnnotator 1.10.4, and the MCC trees were visualized and edited in FigTree 1.4.0.

To elucidate the prevalence of CRF209_cpx, the *pol* gene PR-RT fragments were used to search for archived sequences with genetic similarity in the GenBank using LANL HIV BLAST. The ML tree was constructed to describe the phylogenetic topology and genetic relationships.

### Drug resistance and genotypic co-receptor analysis

The HIVdb program from the Stanford HIV Drug Resistance Database (https://hivdb.stanford.edu/hivdb/by-sequences/) was used to analyze the drug resistance mutations (DRMs) and the sensitiveness to protease inhibitors (PIs), nucleoside reverse-transcription inhibitors (NRTIs), non-nucleoside reverse-transcription inhibitors (NNRTIs), and integrase strand transfer inhibitors (INSTIs). The V3-loop sequences of the NFLG were obtained using the GeneCutter tool. The co-receptor tropism was predicted using the geno2pheno coreceptor 2.5 tool (http://coreceptor.geno2pheno.org/) with a 5% false-positive rate (FPR) threshold and the HIVcoPRED tool (https://webs.iiitd.edu.in/raghava/hivcopred/submit.html).

## Results

### General information of the subjects and identification of URFs

Five individuals were recruited and identified as URFs with discordant subtyping by HIV-1 *pol* gene PR-RT and IN fragments, in Guangzhou from newly diagnosed MSM aged 22–25 between 2018 and 2022. The Sociodemographic and clinical characteristics of these 5 individuals are listed in Table 1.

**Fig. 1:**
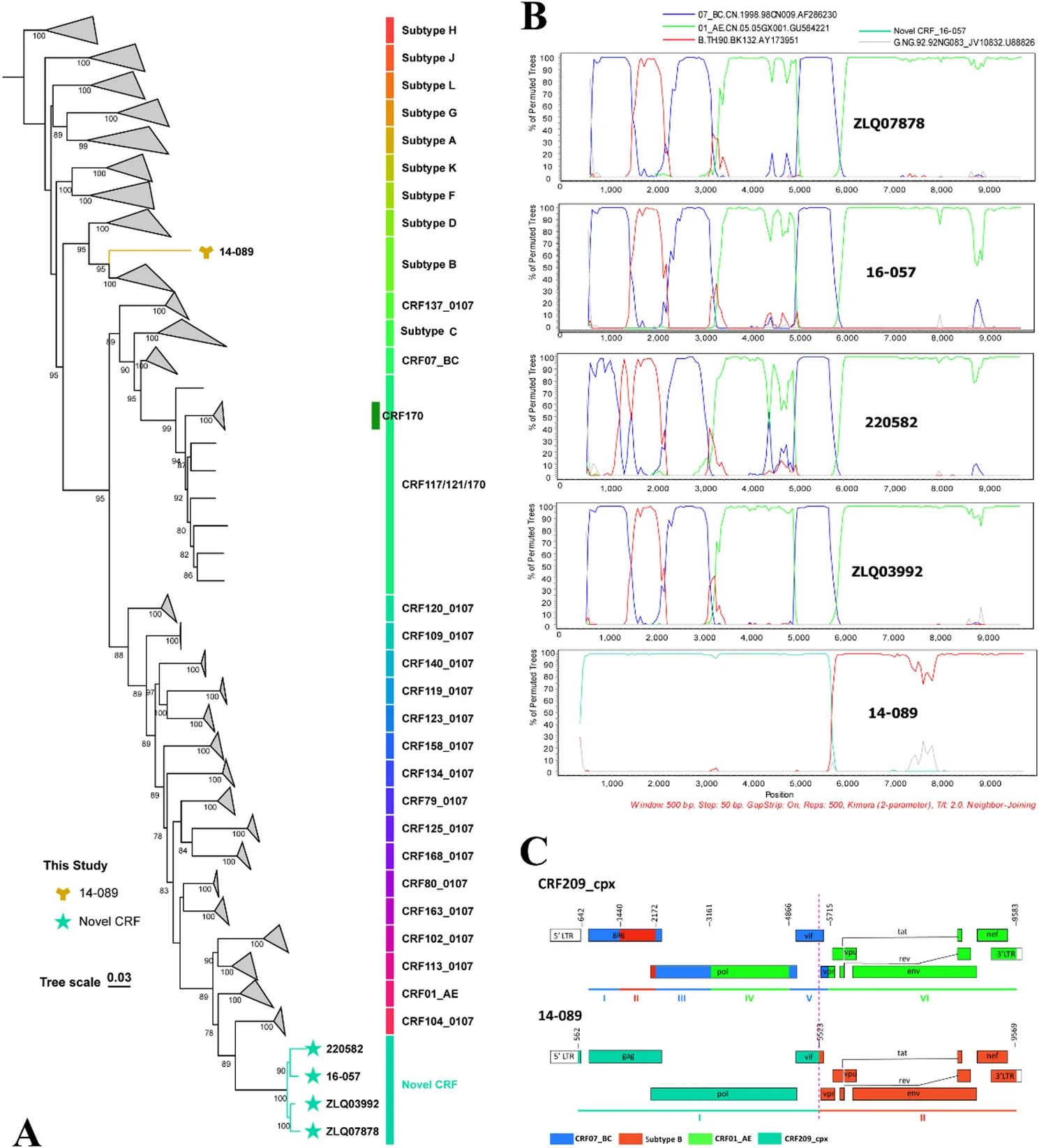
Phylogenetic and recombinant analyses based on the near full-length genome sequences. (**A**) A maximum likelihood phylogenetic tree was constructed using the reference sequences of HIV-1, including 10 subtypes (A-D, F-H, and J-L), CRF01_AE, and CRF07_BC. CRFs composed of CRF01_AE and CRF07_BC have been reported in China. The sequences of CRF209_cpx and URF 14–089 are marked with a star and a wye symbol, respectively. The values at the node represent the percentage of 1000 bootstrap replicates, and the scale bar indicates 10% nucleotide sequence divergence. (**B**) BootScan analysis of candidate CRF209_cpx and URF 14–089, with CRF07_BC, CRF01_AE, subtype B, and G reference sequences. Conditions used in this analysis: window size of 500 bp, step by 50 bp, GapStrip: on, replicates: 500, Kimura (2-parameter), T/t: 2.0. (**C**) Genomic structure of the candidate CRF209_cpx and URF 14–089 with CRF07_BC, CRF01_AE, and subtype B. The genome mosaic maps were generated using the Recombinant HIV-1 Drawing Tool

Four NFLG sequences (ZLQ07878, 16–057, 220582 and ZLQ03992) formed a distinct monophyletic cluster (bootstrap value = 100%) within a clade comprising known inter-CRF recombinants of CRF01_AE and CRF07_BC in the ML tree (Fig. 1A). The phylogenetic cluster was further identified as a novel CRF, designated as CRF209_cpx by LANL HIV database, spanning nucleotide positions HXB2 nt 562 to 9600.

Another individual (ID: 14–089) clustered within subtype B clade with a bootstrap value of 100% (Fig. 1A), exhibited discordant subtyping and topology structures by the 5’ and 3’ terminal halves of NFLG genome, which were distinct from those of established subtypes or CRF clusters.

All sequences have been deposited in GenBank under the accession numbers (PQ539309-PQ539312, PV588241).

### Recombinant breakpoint and subregion phylogenetic analysis

Bootscan analyses (Fig. 1B) confirmed that all five strains recombined among CRF01_AE, CRF07_BC and subtype B. For the four sequences of CRF209_cpx, five breakpoints divided the backbone genome of CRF01_AE into six fragments, I_CRF07_BC_ (HXB2 nt 562–1439), II_B_ (HXB2 nt 1440–2171), III_CRF07_BC_ (HXB2 nt 2172–3160), IV_CRF01_AE_ (HXB2 nt 3161–4865), V_CRF07_BC_ (HXB2 nt 4866–5714) and VI_CRF01_AE_ (HXB2 nt 5715–9600). Otherwise, for URF 14–089 (Fig. 1B and C), the genome was divided by the breakpoint of HXB2 nt 5522 into two fragments, I_CRF209_cpx_ (562–5522 nt) and II_B_ (5523–9569 nt).

**Fig. 2:**
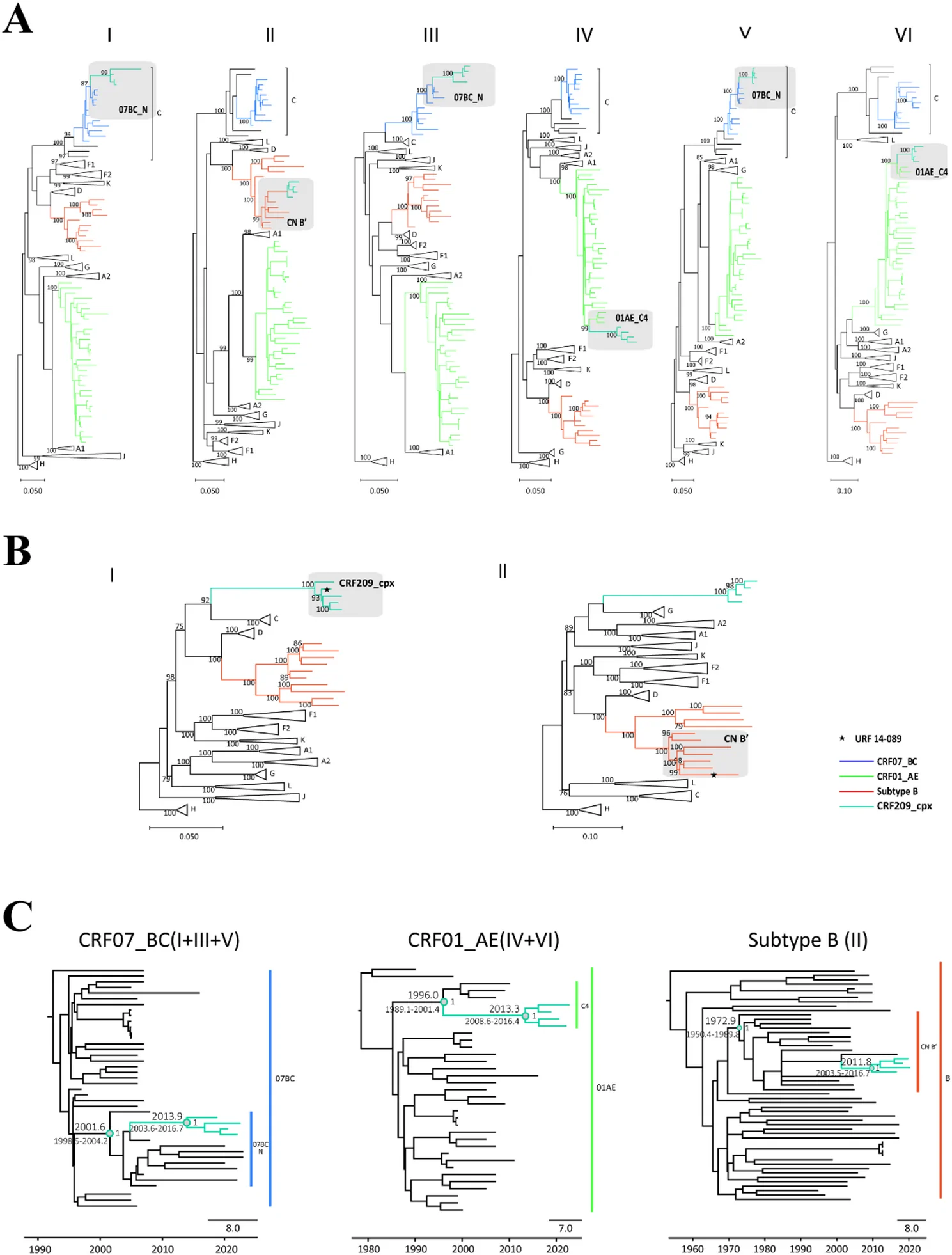
Phylogenetic analysis of individual subregions and Bayesian Coalescent analyses. (**A**) The maximum likelihood (ML) phylogenetic trees of the six subregions of CRF209_cpx. The reliability of the tree branches was assessed using 1000 bootstrap replicates. The scale bar indicates nucleotide sequence divergence. (**B**) The ML phylogenetic trees of URF 14–089. (**C**) The maximum clade credibility trees of the concatenated CRF07_BC subregions (I + III+V), the concatenated CRF01_AE subregions (IV + VI), and the subtype B subregions (II)

The segmental ML trees were reconstructed to ascertain potential parental strains for CRF209_cpx and URF 14–089 (Fig. 2A and B). Fragments I, III and V of CRF209_cpx clustered within CRF07_BC cluster N, with bootstrap value between 87% and 100%, which indicated that this parental strain originated from the CRF07_BC (cluster N) mainly spread among MSM. In contrast, fragments IV and VI of CRF209_cpx clustered within CRF01_AE cluster C4 with bootstrap value of 99% and 100%, respectively, which indicated that this parental strain originated from CRF01_AE (cluster C4) that generally circulated among MSM. Segments II of the CRF209_cpx clustered within subtype B (Chinese B’ clade) sequences. For URF 14–089, segment I clustered within CRF209_cpx and segment II within subtype B (Chinese B’ clade, CN), both with 100% bootstrap value (Fig. 2B).

### The origin of CRF209_cpx and its prevalence

To estimate the origins of CRF209_cpx, Bayesian coalescent analyses were performed to estimate the tMRCA for the concatenated genomic regions derived from CRF07_BC (subregions I + III+V), CRF01_AE (subregions IV + VI) and subtype B (subregions II). The median tMRCA for the CRF07_BC derived segments was 2013.9 [95% highest probability density (HPD): 2003.6–2016.7], whereas those for the CRF01_AE and subtype B derived segments were 2013.3 (95% HPD: 2008.6–2016.4) and 2011.8 (95% HPD: 2003.5–2016.7), respectively (Fig. 2C). Therefore, it is estimated that CRF209_cpx might have recombined and emerged between 2011 and 2013.

**Fig. 3:**
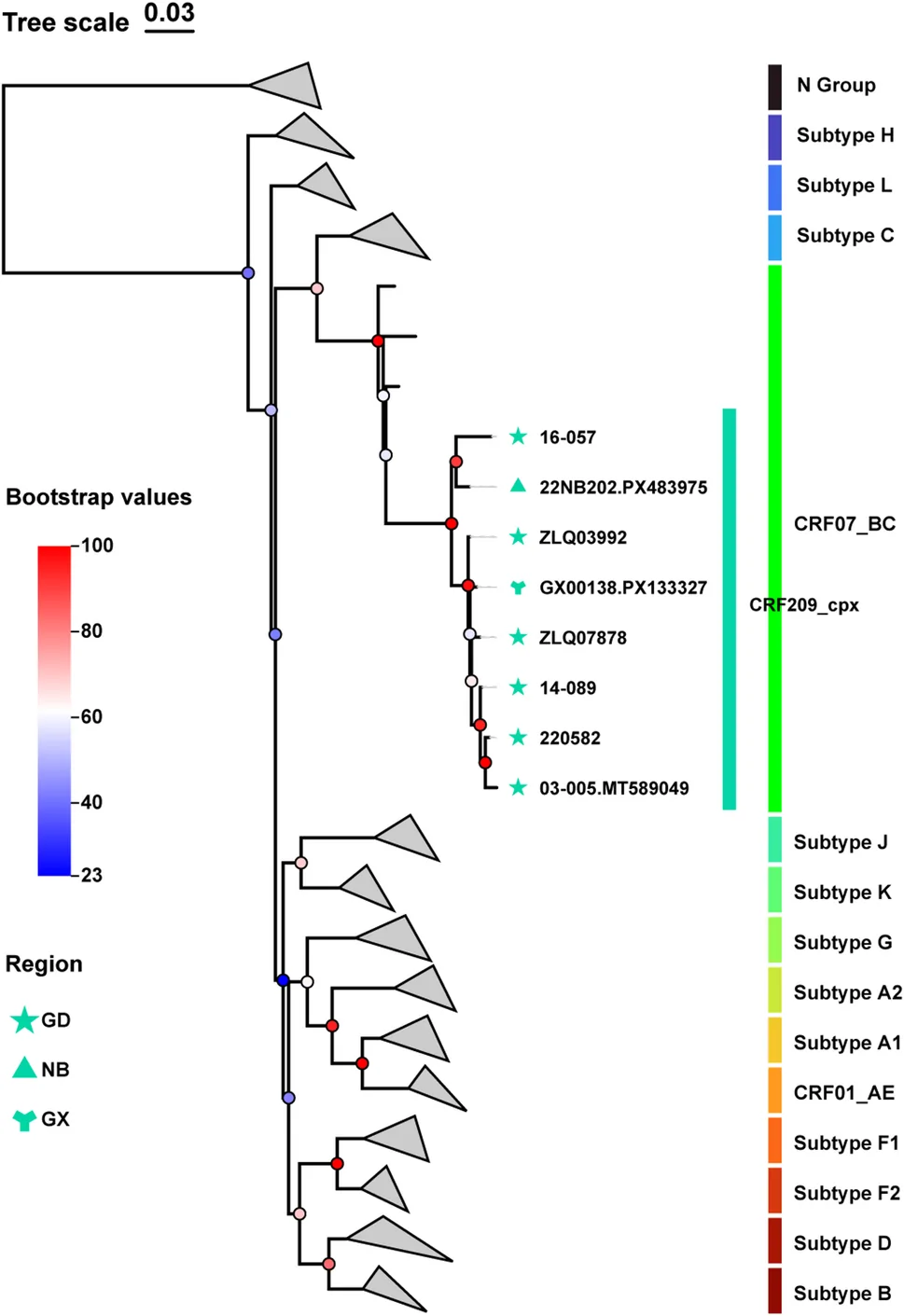
Phylogenetic analysis of the PR-RT gene region (HXB2 nt 2241–3326) of CRF209_cpx and sequences searched by BLAST and downloaded from the LANL HIV database

The PR-RT region of the *pol* gene (HXB2 nt 2241–3326) was used to retrieve the top ten genetical similar sequences in the GenBank. The ML phylogenetic analysis revealed that three sequences of 22NB202 (Accession No. PX483975, sampling in 2022 from Ningbo, Zhejiang) GX00138 (Accession No. PX133327, sampling in 2020 from Nanning, Guangxi) and 03–005 (Accession No. MT589049, sampling in 2018 from MSM in Guangzhou, Guangdong) clustered with five sequences in this study (Fig. 3). Bootscan analyses indicated that the three archived *pol* gene sequences carried the same breakpoints similar to those of CRF209_cpx, implying that CRF209_cpx may have spread outside Guangdong Province.

### Prediction of drug resistance and coreceptor usage

No pretreatment drug resistance mutation was identified in the five individuals, for PIs, NRTIs, NNRTIs and INSTIs. Coreceptor tropism prediction based on the V3 loop sequences indicated that four strains of CRF209_cpx were CXCR4-tropic, whereas URF 14–089 was R5/X4 dual-tropic.

## Discussion

Recently, a significant emergence of novel CRFs and URFs has been documented in Guangdong, indicating an active HIV epidemic and transmission dynamics among populations at high risk of HIV [7, 14]. Here, we reported a novel CRF209_cpx, the second complex recombination in Guangdong, involving recombination among CRF01_AE, CRF07_BC and subtype B from young MSM in Guangzhou, between 2018 and 2022.

In Guangdong, subtype B was the major circulating strain before 2000, and the HIV-1 epidemic has been predominantly driven by CRF01_AE and CRF07_BC since 2006, which together account for more than 70% of infections [5–6]. The worldwide rise in the prevalence of recombinant strains is attributed to multiple diversity with active sexual behavior and cross population contacts, inducing consequent coinfections or superinfection between diverse subtype populations [4, 15].

Guangdong is the region severely affected by HIV-1 CRF07_BC, CRF01_AE and subtype B [7]. Co-circulation with multiple genotypes facilitates the generation of HIV-1 recombinants. The MSM population was a hotspot for viral recombination due to dual infection and superinfection, which have been frequently reported [4, 7, 16]. Previously, phylogenetic and genetic distance analyses revealed that CRF01_AE has been evolved into 11 clusters, and CRF07_BC into CRF07_BC-N and CRF07_BC-O clusters in China, whereas subtype B has developed into four distinct lineages: B’, BJ-B, Pan-B, and TW-B in China [10–11]. Phylogenetic inference indicated that the parental segments of all five individuals in this study were genetically derived from CRF01_AE cluster C4, CRF07_BC cluster N and B’. Epidemiologically, CRF01_AE cluster C4 and CRF07_BC cluster N were primarily transmitted among MSM in China [7, 17]. The B’ strains were first identified among the injecting drug users (IDUs) in Yunnan in 1989, and later spreading to former plasma donors and finally to the general population and MSM via sexual contact [18]. All five individuals in this study were infected through MSM, and the phylogenetic inference of the origin for the parental strains supported this fact. Therefore, it is speculated that these five CRF01_AE/CRF07_BC/subtype B recombination strains likely originated locally within the MSM population, rather than being introduced from other population at high risk.

Owing to the high risk for HIV-1 infection with unprotected sexual contact, multiple sexual partners, and the co-circulation of multiple CRFs and subtypes, many URFs and CRFs were frequently reported among the MSM population [4, 7, 19]. Several recombinant forms involving CRF01_AE, CRF07_BC and subtype B have been documented in China, with cases identified in Guangxi (one URF sequence), Hebei (two URF sequences), Anhui (one URF sequence), and Guangdong (eight CRF162_cpx sequences).

We selected the *pol* gene PR-RT region of CRF209_cpx and the most similar sequences were searched and downloaded using the BLAST tool from the LANL HIV-1 database. The ML phylogenetic analysis of the PR-RT region (HXB2: 2241–3326 nt) (Fig. 3) revealed that the three sequences of 22NB202, GX00138 and 03–005 clustered with the five sequences in this study. The three partial *pol* sequences carried the similar breakpoints to CRF209_cpx, therefore we speculate that CRF209_cpx may not be confined to Guangdong Province.

All 5 strains were predicted to utilize the CXCR4 co-receptor or R5/X4 dual tropisms. Previous clinical cohort studies have shown that X4 and dual tropic viruses are rarely observed during the early stage of infection. Patients infected with X4 or R5/X4 dual-tropic strains exhibited significantly faster decline in CD4 + T cells, rapid disease progression and a higher risk of clinical events (e.g. opportunistic infections) than those infected with R5-tropic strains. Therefore, it is urgent to reinforce HIV-1 molecular epidemiological investigations to curb ongoing transmission.

In summary, a novel CRF209_cpx was identified through recombination from CRF01_AE, CRF07_BC and subtype B and is estimated to have originated between 2011 and 2013 among the MSM population in Guangdong Province. Furthermore, we detected a novel third-generation URF between nascent CRF209_cpx and subtype B. These facts imply that the HIV epidemic is active and complicated among MSM in Guangdong, China. Molecular epidemiological surveillance and precise control measures should be reinforced for this population.

### Limitations of the study

This study presents several obvious limitations. First, the small sample size of four CRF209_cpx cases weakened our epidemiological inference. However, the four individuals plus the fifth cases of URF CRF209_cpx/B were identified during the surveillance of pretreatment drug resistance. Second, the HIV-1 viral loads at diagnosis were unavailable for the five cases, making it difficult to correlate viral genetic features and tropism to clinical disease progression. Third, viral tropism was merely predicted via computational simulation instead of phenotypic experimental verification, which brought uncertainty to relevant genetic classification. Furthermore, the speculation that CRF209_cpx has spread outside Guangdong Province is only supported by three partial *pol* gene sequences covering PR-RT region retrieved from LANL HIV database, lacking more detailed epidemiology information and the near full-length genome sequences. The segmental genetic information cannot bring firm evidence to validate the cross-regional transmission tendency. Here, we just give our preliminary judgement and conjecture of CRF209_cpx spread.

Finally, our observations offer fundamental evidence for monitoring HIV-1 recombinant variants in Guangdong Province, and molecular epidemiology should be well performed, to describe the spreading characteristics of CRF209_cpx.

## Supplementary Information

Supplementary Material 1.

## Data Availability

All sequences have been deposited in GenBank under the accession numbers (PQ539309-PQ539312, PV588241).
