# Supplementary material for: Identification of a novel HIV-1 circulating recombinant form (CRF209_cpx) and its descendant unique recombinant form (URF) CRF209_cpx/B among MSM in Guangdong, southern China
Source: Virol J. 2026 Jun 28;23:212. doi: 10.1186/s12985-026-03237-8 (PMC13576128; doi:10.1186/s12985-026-03237-8)
Supplement: Supplementary file 1 [file 12985_2026_3237_MOESM1_ESM.docx]

**Supplementary Table 1** The best-fit models chosen and applied by IQ-TREE for the maximum likelihood phylogenetic tree in this study

| **Tree name** | **Figures in the manuscript** | **Best-fit model** |
| --- | --- | --- |
| NFLG-based tree | Fig 1A | GTR+F+R6 |
| Subregion I tree of CRF209_cpx | Fig 2A | GTR+F+I+G4 |
| Subregion II tree of CRF209_cpx | Fig 2A | GTR+F+I+G4 |
| Subregion III tree of CRF209_cpx | Fig 2A | GTR+F+R4 |
| Subregion IV tree of CRF209_cpx | Fig 2A | GTR+F+R4 |
| Subregion V tree of CRF209_cpx | Fig 2A | GTR+F+R4 |
| Subregion VI tree of CRF209_cpx | Fig 2A | TVM+F+R6 |
| Subregion I tree of URF 14-089 | Fig 2B | GTR+F+R4 |
| Subregion II tree of URF 14-089 | Fig 2B | GTR+F+R5 |

**Abbreviation:**

GTR, General Time Reversible; F, Empirical Base Frequency; TVM, Transversion Model; R4, Free Rate Model with 4 categories; R5, Free Rate Model with 5 categories; R6, Free Rate Model with 6 categories; I, Proportion of Invariable Sites; G4, Gamma Distribution with 4 rate categories
